# Migration, labor and women’s empowerment: Evidence from an agricultural value chain in Bangladesh

**DOI:** 10.1016/j.worlddev.2021.105445

**Published:** 2021-06

**Authors:** Alan de Brauw, Berber Kramer, Mike Murphy

**Affiliations:** a Markets, Trade, and Institutions Division, International Food Policy Research Institute (IFPRI), 1201 Eye Street, NW, Washington, DC, USA; b Mara House, International Livestock Research Institute (ILRI), Naivasha Road, Nairobi, Kenya

**Keywords:** Rural, Labor, Agriculture, Migration, Gender, Bangladesh

## Abstract

As a substantial portion of the rural labor force migrates to urban areas, it is commonly assumed that women could take over traditionally male tasks in agricultural production, with potentially empowering outcomes for women. We study how changes in the supply of labor may influence female labor participation and empowerment outcomes. Using a detailed panel dataset on jute producers in the delta region of Bangladesh, we test whether out-migration of household members and perceived labor shortages are associated with the share of household and hired labor performed by women, and women’s empowerment. When a household experiences reduced household or hired labor supply, we observe a relatively larger use of female household labor, but a reduced share of female hired labor. We also find that reduced labor supply is not associated with significant reductions in gender wage gaps, or enhanced women’s empowerment. These findings suggest that given existing gender norms, male and female labor are not perfect substitutes for one another, and as a result, male outmigration is not associated with improved outcomes for women in cash crop production in the short run. Our results demonstrate a need for better understanding of the role of gender in rural labor markets, particularly in contexts of rapid urbanization.

## Highlights

• We study changes in rural labor supply and their association with female empowerment.
• Labor scarce households see increased work by women only in traditionally female tasks.
• Reduced labor supply is not associated with a reduction in gender wage gaps.
• Households with female migrants have enhanced empowerment of other household women.
• Male migration is not linked to enhanced outcomes for women in the short run.

## 1 Introduction

Gender has historically played an important role in shaping the division of labor in agricultural production, and this, in turn, has been shown to have persistent effects on contemporary gender norms in industrialized societies (Alesina et al., 2013, Boserup, 1970). Globally, women have increasingly taken on ‘visible’ public roles in the economy, but without a corresponding increase in the extent to which men participate in care work within households (Evans, 2016). As urbanization occurs, and a significant share of the rural labor force migrates to urban areas, it is commonly assumed that women could take over traditionally male tasks in agricultural production (i.e. Slavchevska, Kaaria, & Taivalmaa, 2019). Such changes may lead individuals to update their beliefs around women’s abilities to perform certain tasks and empower women by transforming gender norms. Conversely, an increased role in agricultural production may simply increase women’s workload, without necessarily empowering women or enhancing their wellbeing (Pattnaik, Lahiri-Dutt, Lockie, & Pritchard, 2017). The relationship between migration of the rural labor force into cities, increased female participation in agricultural activities, and women’s empowerment status is therefore unclear, at least in the short run.

In this paper, we explore how changes in labor supply may influence female labor participation and empowerment outcomes in the short run. From an agricultural perspective, using a collective household model (e.g. Chiappori, 1992), there are four possible reactions to changes in the local rural labor supply. Consider first that men leave the specific household. In that case, women could take over their agricultural tasks. Second, the household could hire substitute labor, though hired labor is an imperfect substitute for household labor (Benjamin, 1992, Singh et al., 1986). Third, households could substitute capital for labor, at least for tasks for which machine rental is possible. And fourth, if they do not fear land expropriation (e.g. Jacoby, Li, & Rozelle, 2002), then they can reduce the intensity of their cultivation. If households are not reducing cultivation intensity but labor is becoming scarcer, the cost of hiring labor can increase. Then households that had used hired labor for tasks may also find it advantageous to either allocate those tasks to women, substitute capital for labor, or reduce the intensity of their cultivation.

To answer our research question, we analyze data from a context where migration and gendered norms around agricultural production are both prevalent: smallholder farmers in the southern delta of Bangladesh. Using a panel survey of jute producers in the region, we study how changes in gender gaps in labor participation, wages and empowerment are associated with two variables that could be indicative of changes in labor supply: whether a household reports an adult member migrating during the survey period (as an indication of reduced labor supply) and whether the household reports having faced increased difficulties finding enough labor for their jute production during that period, as reported by the primary agricultural decision-maker in the household.^1^ As our empowerment indicator, we use the project-level Women’s Empowerment in Agriculture Index (pro-WEAI) (Malapit et al., 2019), which was administered to the same male and female respondents within the household during two follow-up survey rounds.

Analyzing detailed gender-disaggregated data on labor used for jute production and post-harvest activities, we first establish that the division of labor in jute in our study area follows strong gender norms. Whereas male labor is used for a wide range of tasks, female labor is used mainly for post-harvest processing activities typically carried out at the homestead. For households reporting migration, we observe an overall increase in the share of labor performed by household females and a reduction in the share by hired females. Female laborers are paid lower wages than men, even when focusing on activities where the use of female labor is prevalent, and men and women perform the same tasks. This gender wage gap does not decrease when households have likely faced a reduction in labor supply. These findings suggest that in the presence of strong gender roles in agricultural production, female labor is not treated as a substitute for male labor, so changes in household labor force composition do not necessarily create opportunities to empower women in agriculture.

This paper contributes to the literature in several ways. First, we contribute to an empirical literature which explores how migration is associated with allocation and investment decisions by sending households. Böhme (2015) uses state-level U.S. GDP growth rates to instrument for the relative returns to migration among households with migrants in rural Mexico and finds an increase in agricultural investment among households with migrants. Similarly, using an instrumental variables approach, De Brauw (2010) finds a shift away from labor intensive crops among rice producing households with migrants in Vietnam. Mergo (2016) exploits data on participation in the US visa diversity lottery program by Ethiopian household members and finds that households with migrants increase food consumption and energy expenditures, while those with specifically male migrants also increase investment in water and sanitation improvements. Closest in focus to our study, Wang, Rada, Qin, and Pan (2014) test how households allocate labor inputs in response to out-migration, finding that households with migrants meet labor maintain levels of labor for rice production by shifting family labor from non-rice crops and off-farm activities.

Second, we explore the role of migration in relation to gendered labor allocation for agriculture in a novel setting. Outside of agriculture, several studies explore the labor dynamics among non-migrating women in contexts where migrants are predominantly male. For example, Lokshin and Glinskaya (2009) find that male migration reduces female labor force participation using a nationally representative data set from Nepal, though they note the effect is weaker in households with large landholdings. Also in Nepal, Holmelin (2019) more subtly finds that women in households with male migrants take on more agricultural labor, and temporarily take over decisions about farm management. Mendola and Carletto (2012) study male-dominated migration from Albania and find a negative association with formal female wage employment in households with migrants. Similarly, Kan and Aytimur (2019) find no increases in female labor force participation by women from migrant sending households in Tajikistan and posit that norms around employment outside the household may prevent women from taking on new roles.

Within agriculture, evidence on gendered labor allocation is predominantly from studies in China. Mueller, Kovarik, Sproule, and Quisumbing (2015) provide a review, with the common finding of an increased role in agricultural production by women and children in households where the adult male has migrated (Chang et al., 2011, de Brauw and Mu, 2011, Mu and van de Walle, 2011). Since norms about women’s role in agricultural production– and labor force participation more generally– are determined by cultural context, it is important that the evidence base on the topic is drawn from a diverse range of settings to identify both commonalities and differences in outcomes for women. Focusing on jute, an important cash crop in Bangladesh, we find an increase in the relative share of household labor undertaken by women, but a decrease in labor by hired women, and little evidence to suggest that women are taking on new roles in agricultural production.

Third, a key contribution of our paper is to demonstrate the value of linking detailed gender-disaggregated data on agricultural activities with measures of female (and male) empowerment within households. We collect both data on labor use by male and female, hired and household workers for each stage of agricultural production for their primary cash crop, and detailed individual-level measures of empowerment within agriculture using the pro-WEAI, a new, internationally validated, measure of women’s empowerment designed for project use. We combine traditional economic measures of welfare for men and women– in terms of their time worked and compensation received– with data on their perceptions of their own individual and collective agency. We hope to encourage future research to incorporate measures of agency within their theory of change, as a complement to measures of economic wellbeing.

## 2 Data and methods

### 2.1 Setting

We use data collected from smallholder household surveys conducted in four districts of the southern delta region in Bangladesh (Faridpur, Jhenaidah, Madaripur and Narail). Data collection was conducted as part of the Agricultural Value Chains (AVC) program in Bangladesh, a project which aimed to improve agricultural incomes and food security through a value chains development approach. Details of the project are described in de Brauw, Kramer, Martinez, and Murphy (2018). We focus exclusively on households who produce jute as their primary crop, as it was one of the crops targeted by the value chains development program.

As an agricultural commodity, jute refers to the vegetable fibers extracted from the shrub species *Corchorus olitorius* and *Corchorus capsularis*^2^ which are primarily used as a natural fiber in the production of textiles. Jute has long been produced in Bangladesh, playing a significant role in the globalization of trade in the 19th century where it was used to produce coarse bags for packaging commodities for shipment.^3^ As such, jute is a salient crop for our study. As a crop that has been widely cultivated in the region for a long period of time, it presents an opportunity to examine established gender norms around agricultural production. Jute is a wholly commercial crop, so it is unlikely to be affected by changes in household composition through the demand channel, in contrast to horticultural produce and livestock which may be used either for sale or household consumption, though as an export crop it can also leave farmers potentially more exposed to shifts in international trade.^4^

### 2.2 Sample selection

To establish a representative sample of jute producing households, we listed all farming households in fifty jute producing villages prior to the baseline survey. To be eligible for selection, households had to report intending to plant jute as their primary crop in the coming season and cultivate at most 500 decimals (2.02 ha), though most farmers cultivated substantially less land.^5^

For those meeting these sampling criteria, twenty households from each village were randomly selected for an interview in 2016 to create a total sample of 1000 households. This sample was expanded to include ten additional farmers from each village in 2017, bringing the total sample size to 1500.^6^ We exclude households who did not report producing jute throughout the study period, as well as those who could not be located or refused to participate in one or more interviews. As a result, our analysis sample consists of 1410 households (equivalent to a 6% attrition rate): 924 from the original 2016 sample and 486 from the additional 2017 sample. Respondents were interviewed at their dwelling around the end of the primary agricultural season (March-April) up to three times (2016, 2017, and 2018). Our analysis focuses on data from the interviews conducted in the 2017 and 2018 survey rounds to maximize the available number of observations.

### 2.3 Survey instruments

The survey consisted of two separate forms. The respondent for the main form was the primary agricultural decision-maker in the household (who was generally male) and focused on agriculture, with detailed modules on jute production, production of other crops, livestock holdings, allocation of labor and other inputs, as well as other household level outcomes not related to agriculture. The labor module is the primary source of data for analysis. It was designed around the specific stages of jute production, comprising eleven stages from land preparation through transportation and marketing of jute fibers. For each stage, we collected information on the type and duration of labor use for both household and hired laborers, disaggregated by gender within each category. We also collected data on wages paid to hired laborers and perceived challenges in finding labor.

A secondary form was administered at the individual level within each household to the spouse of the primary agricultural decision-maker (typically female). It included questions on household members, including migration, and household consumption. For the additional sample, both the main form and this secondary form also included a series of modules comprising the project-level Women’s Empowerment in Agriculture Index (pro-WEAI), which collected detailed data on a range of domains of empowerment (Malapit et al., 2019). Hence, for the additional sample, we have responses to pro-WEAI modules from both male and female household respondents. Interviews were administered in private by an enumerator of the same gender as the respondent, to ensure respondents could share information on potentially sensitive topics while preserving their privacy. Table 1 provides an overview of the data collected for both samples.

**Table 1:** Available data categories for analysis, by sample and survey year.

Year	Original Sample			Additional Sample		
	Labor	Migration	Pro-WEAI	Labor	Migration	Pro-WEAI
2016	✓			No survey		
2017	✓	✓		✓		✓
2018	✓	✓		✓	✓	✓

### 2.4 Variable definitions

Our analysis focuses on two explanatory variables that measure changes in the amount of labor available to households in our sample over the survey period.^7^ The first is a variable indicating whether a household reported that one or more of its members had migrated since the previous survey round. If an individual who had been present in the previous survey round was not present, the individual responding to the secondary form was asked why that person had left. If the person had left the household to migrate either temporarily or permanently, this variable was coded as one, and zero otherwise.^8^ Note that our analysis is therefore a within-household comparison of changes associated with migration over the study period, rather than a between household comparison of migrant and non-migrant households. We also disaggregate this variable by gender, to create two variables employing the same definition: whether the household reported one or more male (or female) members having migrated.^9^

The second key explanatory variable is an indicator for reported availability of hired labor. As part of the main survey labor module, after providing estimates of wages paid to male and female laborers, the primary decision-maker was asked whether they had experienced difficulty finding enough labor for a given jute production stage in the preceding agricultural season. We aggregate this variable across activities, so the indicator takes the value one if the farmer reports having faced difficulties finding labor for jute production during at least one stage of production, and zero otherwise.

For outcomes, we consider two groups of variables: labor-related outcomes, which are available for the full sample, and empowerment measures constructed using responses to the Pro-WEAI modules, which are only available for the additional sample. All labor outcomes are disaggregated by gender and reported separately for each activity in jute production.^10^ We construct indicators of female labor participation as the amount of female household labor as a share of the total amount of household labor, the amount of female hired labor as a share of the total amount of hired labor, and the amount of female labor as a share of the total amount of labor used. We aggregate these labor shares by summing across activities, whereas wages are aggregated across activities by taking the mean wage across activities for which a female (or male) worker was hired. Aggregated expenditures are divided by the area (in decimals) on which the household cultivated land to facilitate comparisons between farmers with differently sized farms.

For the pro-WEAI outcomes, the survey modules capture information on three domains of empowerment (3DE). The domains are intrinsic agency (one’s own power); instrumental agency (power to do); and collective agency (power as part of a group). Within these three primary domains, questions are divided into twelve sub-domains.^11^ For each of these sub-domains an adequacy score is computed to determine an individual’s level of empowerment within that sub-domain.^12^ The individual is then assigned a binary score for each sub-domain (which takes the value one if the adequacy score for that sub-domain is sufficiently high, and zero otherwise). If the mean value of these scores is greater than or equal to 0.75, or in other words if their level of empowerment is adequate in at least nine of the twelve sub-domains, a respondent is considered empowered.

For our empowerment outcomes we use both an indicator variable for whether the respondent is considered empowered overall (i.e. whether the respondent’s level of empowerment is adequate in at least 75 percent of the sub-domains), and a continuous variable that represents the proportion of the twelve sub-domains in which the respondent is considered adequately empowered. Combined, these two variables allow us to compare both changes in the share of those meeting the overall empowerment threshold, and changes in the share of sub-domains in which respondents are considered empowered.

### 2.5 Estimation

For our primary specifications of interest, we estimate two regressions with household fixed effects.^13^ We first estimate the change in a given outcome *Y* associated with a change in our two main explanatory variables: *Migrant*, an indicator variable which takes the value 1 if the household reports at least migrant at survey period *t* and 0 otherwise, which we interpret as a proxy for reduced household labor availability; and *Scarcity*, an indicator variable which takes the value 1 if a household reports difficulty finding labor for period *t* and 0 otherwise, which we interpret as a proxy for reduced supply of hired labor.^14^ The model is estimated as a pooled regression, with household and survey round fixed effects (δ and γ), a dummy variable taking on a value of one for the additional sample in the 2017 round and zero otherwise (represented by ζ), a control variable for the area on which a farmer planted jute, and the error term ∊:

(1) $$Y_{\mathrm{it}}=\alpha +\beta_{1}{\mathrm{Migrant}}_{\mathrm{it}}+\beta_{2}{\mathrm{Scarcity}}_{\mathrm{it}}+\beta_{3}area_{\mathrm{it}}+\delta_{i}+\gamma_{t}+\zeta_{\mathrm{it}}+{∊}_{\mathrm{it}}$$

We also estimate a variation of this specification, disaggregating *Migrant* by gender, so *MaleMigrant* (*FemaleMigrant*) takes the value 1 if the household reports that at least one male (female) household member migrated out of the household between survey period *t* − 1 and *t*, and 0 otherwise. This equation is:

(2) $$Y_{\mathrm{it}}=\alpha +\beta_{1}{\mathrm{MaleMigrant}}_{\mathrm{it}}+\beta_{2}{\mathrm{FemaleMigrant}}_{\mathrm{it}}+\beta_{3}{\mathrm{Scarcity}}_{\mathrm{it}}+\beta_{4}area_{\mathrm{it}}+\delta_{i}+\gamma_{t}+\zeta_{\mathrm{it}}+{∊}_{\mathrm{it}}$$

For individuals in the additional sample, we do not observe migration in the first survey round. As a result, we assign these observations zero for migration indicators for our main specification. Therefore, we cannot estimate the pooled specification for empowerment outcomes, since these outcomes are only observed among the additional sample. Instead, for this set of outcomes, we specify a static model, where we regress an outcome in the second period (*t = 1)* on reported migration in the second period, and control for the lag of the outcome reported in the first period. Since we observe reported labor scarcity in both periods, we take the difference between the second and first period, giving:

(3) $$Y_{i,t=1}=\alpha +\beta_{1}{\mathrm{Migrant}}_{i,t=1}+\beta_{2}\left({\mathrm{Scarcity}}_{i,t=1}-{\mathrm{Scarcity}}_{i,t=0}\right)+\beta_{3}\left(area_{i,t=1}-area_{i,t=0}\right)+Y_{i,t=0}+{∊}_{i}$$

As in (1), we additionally disaggregate the migration indicator variable by gender:

(4) $$Y_{i,t=1}=\alpha +\beta_{1}{\mathrm{MaleMigrant}}_{i,t=1}+\beta_{2}{\mathrm{FemaleMigrant}}_{i,t=1}+\beta_{3}\left({\mathrm{Scarcity}}_{i,t=1}-{\mathrm{Scarcity}}_{i,t=0}\right)+\beta_{3}\left(area_{i,t=1}-area_{i,t=0}\right)+Y_{i,t=0}+{∊}_{i}$$

We cluster standard errors at the village level across all specifications. We also control for the area planted with jute, as it will be an important time-varying determinant of our outcome variables and would not be captured by household fixed effects.

An important qualifier to our results is that they are by nature descriptive, rather than causal. Since the decision by a household member (or members) to migrate is not plausibly exogenous, we are not able to construct the counterfactual effect of what would have happened had an individual remained (or conversely migrated from a household not reporting migrants). Our aim is rather to describe observable changes in labor allocation and empowerment status within households with migrants versus those without, as these changes have important implications for thinking about policies around gender, migration and women’s empowerment in agriculture.

## 3 Descriptive statistics

### 3.1 Respondent and household characteristics

Table 2 presents summary statistics on the sample households and their agricultural production. The person identified as the main respondent was almost exclusively male—only 4% of household identified a woman as the primary decision-maker on jute production. Almost all (95%) are married. They are typically middle aged, with an average age of 48 years, compared to migrants who are generally young, with an average age of 21. Migration in our sample is therefore generally a change in the available pool of potential household laborers available to a decision-maker, rather than a change in the individual making the decisions. These available laborers are roughly balanced in terms of gender, with a slightly higher mean number of available women than men.

**Table 2:** Primary respondent and farm characteristics.

*Panel A: Primary respondent characteristics*					
	Mean	SD	Min	Max	N
Is female	0.04	0.20	0.00	1.00	1410
Age	48	12	19	99	1410
Is married	0.95	0.21	0.00	1.00	1410
Is Muslim	0.69	0.46	0.00	1.00	1410
Is Hindu	0.31	0.46	0.00	1.00	1410
Is literate	0.50	0.50	0.00	1.00	1410
Has completed primary school	0.40	0.49	0.00	1.00	1410
Has completed secondary school	0.09	0.29	0.00	1.00	1410

*Panel B: Farm characteristics*					

	Mean	SD	Min	Max	N

Jute area cultivated (decimals)	99	65	10	460	1410
Jute harvested quantity (kg)	1064	821	20	8800	1410
Jute sales revenue (USD)	521.66	405.79	8.80	3470.66	1410
Number of non-jute crops	4	2	0	16	1410
Non-jute sales revenue (USD)	549.51	586.09	0.00	3274.10	1410

The majority of households in our sample are Muslim (69%), with the remaining portion practicing Hinduism. Respondents have had limited access to formal education. Half the sample is illiterate, while only 40% report having completed primary schooling, and less than 10% report having completed secondary school. The average household in the sample has between four and five members, of which approximately three are of working age (15 years or older).

The average household cultivated an area of 99 decimals of jute (0.99 acres) in 2017. While the farmers in our sample are all smallholders, there is nonetheless considerable variation in the reported area planted (between 10 and 460 decimals). In addition to growing jute, most households produced other crops, principally rice and vegetables, both for sale and consumption. The mean revenue from jute sales in 2017 was $521.66, slightly less than the total mean amount of revenue received from all other crops ($549.51).

All households employ household labor for jute production, typically of both genders: 98% of households report using male household labor, while 86% of households report using female household labor. Use of hired labor is notably high in the sample, with 96% of households reporting hiring laborers at some point during the season. However, while hiring male labor is common (96% of households), only 53% of the sample reports hiring female laborers.

### 3.2 Gendered labor usage

**Fig. 1:**
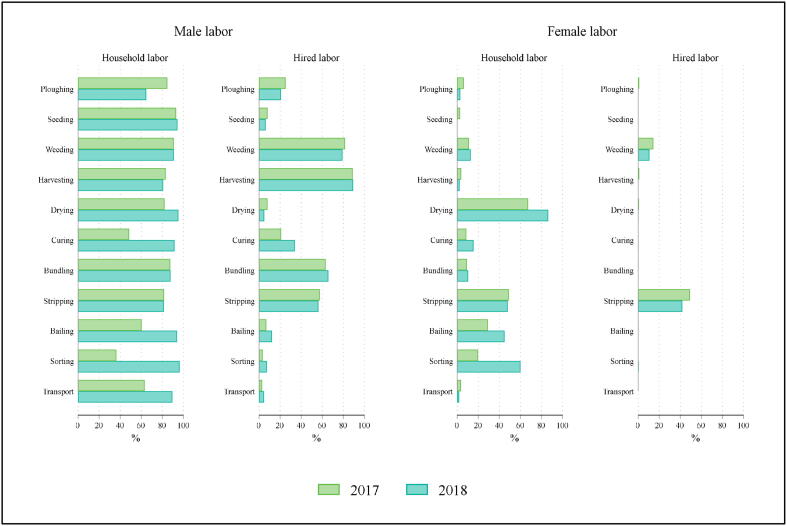
Types of labor used by production activity and gender.

We next consider how households use male versus female labor during different stages of jute production. We organized jute production activities into eleven distinct tasks: pre-harvest activities (ploughing, seeding and weeding); harvesting; processing (drying, curing, bundling, stripping, and bailing); and marketing (sorting and transporting to market). Fig. 1 summarizes the types of labor engaged in each of these tasks.

Whereas male household members are involved across all production and post-production activities, the hiring of male laborers varies by task, with high rates of use reported for both labor intensive (and time sensitive) activities such as weeding and harvesting and some post-harvest activities (bundling and stripping). In contrast, households rarely report using labor by female household members prior to or during harvesting. Female household members are involved primarily in processing activities (stripping and bailing) and in sorting. Hiring of female laborers is essentially constrained to two tasks: weeding and stripping. Female workers, whether household or hired, are almost never involved in transporting and marketing jute. Qualitative research suggests that labor patterns are driven by strong gender-based norms regarding the type of work that women can do, including norms around mobility; post-harvest tasks can be performed in the homestead, in contrast to work in the fields or in transporting jute to market (Rubin et al., 2018).

**Fig. 2:**
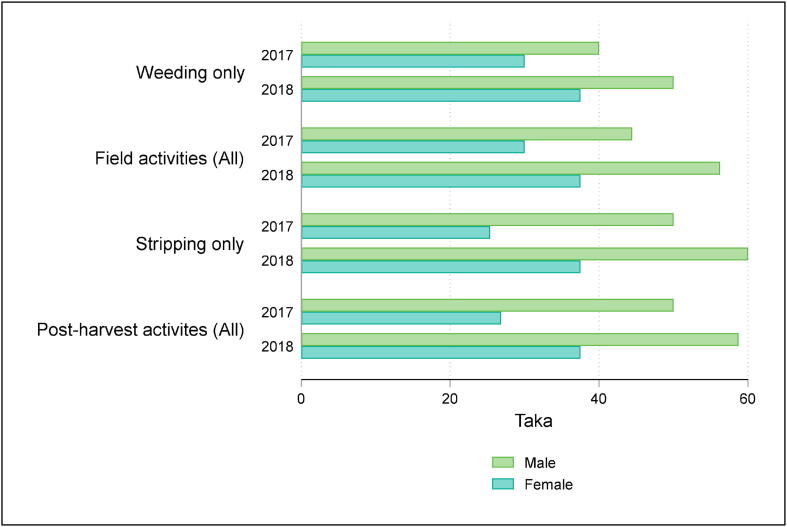
Median hourly wage by production stage and gender.

We next consider gender differences in the outcomes for those providing labor. We first consider wage payments to hired laborers. Fig. 2 shows median wages for male and female hired workers for both survey rounds. We show the two stages for which women are predominantly hired (weeding and stripping) as well as aggregate categories for field activities (ploughing, seeding, weeding and harvesting) and post-harvest activities (drying, curing, bundling, stripping, bailing, sorting and transporting to market).

Across all four categories in both survey rounds, median wages are significantly higher for male than for female workers. In 2017, for weeding, the hourly wage for women was 25% lower than for men, and for fiber stripping, the median wage paid to women was half of what men were paid for the same task. While productivity for field activities could potentially vary by gender, which could help explain a wage gap in field activities, variation in productivity is unlikely for stripping fibers, a fine motor task for which women are typically preferred in the survey area (Rubin et al., 2018). A more likely explanation is that female workers receive less than male workers for equivalent tasks because of lower bargaining power.

**Fig. 3:**
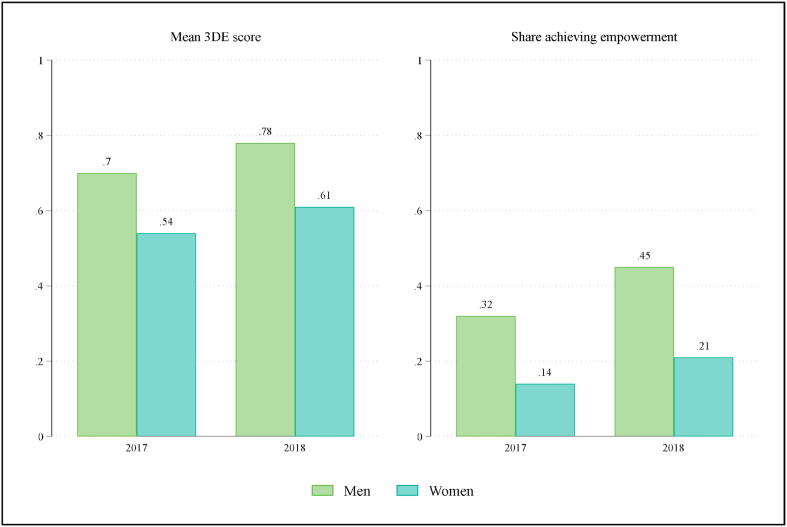
Pro-WEAI status by gender and survey round, additional sample.

Since household members do not earn wages when working on the family farm, we consider an alternative measure of rewards for household laborers: survey respondents’ empowerment within sample households. Focusing on our additional sample, where the pro-WEAI survey instrument was administered, Fig. 3 summarizes the mean 3DE scores and the share of empowered respondents for male and female respondents across the two survey rounds. While empowerment scores increase across the two survey rounds, there is a persistent gap between male and female scores: 14% of women were considered empowered based on the 2017 survey, relative to 32% of men; and 21% based on the 2018 survey, relative to 45% of men.

Overall, we observe strong gender differences in agricultural production in our setting. Gender is predictive of the types of tasks that workers undertake in growing, harvesting and processing jute, the level of compensation that wage workers receive, and the extent to which household members—often providing household labor for jute production—are considered empowered. In the next section, we explore the extent to which these gender gaps change when households report a change in the supply of labor available to them, and whether such changes may represent normative gains for women.

## 4 Findings

### 4.1 Labor allocation

To examine how the use of female labor varies in response to local labor availability, in Table 3 we regress the amount of female labor used, expressed as a share of the total amount of labor used, on our indicator variables for household migration and reported difficulty finding labor during the season (Eqs. (1), (2)). We focus on the share of total labor performed by women to account for variation in the extent or intensity of jute production activities.

**Table 3:** Association between available labor and female labor share.

	Share of total days: Household females		Share of total days: Hired females		Share of total days: Females	
	(1)	(2)	(3)	(4)	(5)	(6)
At least one migrant	0.018**		−0.022**		−0.004	
	(0.008)		(0.009)		(0.012)	
1 + male migrants		0.022**		−0.022**		−0.000
		(0.011)		(0.010)		(0.015)
1 + female migrants		0.013		−0.012		0.001
		(0.010)		(0.011)		(0.015)
Difficulty finding labor	0.011***	0.011***	0.004	0.004	0.015**	0.015**
	(0.004)	(0.004)	(0.007)	(0.007)	(0.007)	(0.007)
Mean male HH adults (2017)	1.5	1.5	1.5	1.5	1.5	1.5
Mean female HH adults (2017)	1.6	1.6	1.6	1.6	1.6	1.6
Mean outcome (2017)	0.11	0.11	0.10	0.10	0.21	0.21
Mean outcome (2018)	0.12	0.12	0.04	0.04	0.17	0.17
p-value: Male = Female		0.580		0.461		0.946
Observations	1410	1410	1410	1410	1410	1410

Notes: Ordinary least squares regression using pooled sample with household-level fixed-effects. Controls are included for the area cultivated with jute by the household, and an indicator variable that takes the value 1 if an observation is of an ‘additional’ sample household in 2017, and zero otherwise. Standard errors are clustered by village. *,**, *** refer to significance at the 10%, 5% and 1% level respectively.

In columns (1) and (2), the dependent variable is defined as the number of days that female household members worked on jute production as a share of the total number of days of labor used in jute production. Columns (3) and (4) follow the same definition but for hired labor, while columns (5) and (6) combine both female labor used (household and hired labor) as a share of the total number of labor days used in the entire jute production process.

We find a statistically significant association between households who report at least one member moving out between survey rounds and female labor usage in specifications (1) and (3). The sign of the coefficient varies with the type of labor. Households with migrants report a modest increase in the use of female household members’ labor as a share of total labor days (1.8 percentage points). For hired labor, the correlation is negative: households with migrant members report hiring less female labor as a share of total labor days (2.2 percentage points). Specifications (2) and (4) suggest that the changes are statistically significant only for households with male migrants, but these modest changes are not significantly different from the changes in labor composition in households with female migrants. In other words, in this context, male migration does not appear to be associated with increased labor market opportunities for women. In fact, our findings suggest that male out-migration is associated with reduced demand for female hired labor.

Our reported measure of difficulty in finding labor is more likely to reflect perceived scarcity of hired labor. Here, the association between labor scarcity and the share of labor performed by women is positive across specifications, though the coefficient is not statistically significant in specifications (3) and (4); households reporting increased labor scarcity use more labor by female household members, but surprisingly, we observe no shifts in the relative use of female hired labor when households perceive increased difficulties finding labor.

In Table 4, we disaggregate the share of labor performed by women by production stage. We do so by separating the two activities for which female labor use is most prevalent (weeding and stripping) and aggregating activities performed on plots (field activities, including weeding; Panel A) relative to activities performed following harvest (post-field activities, including stripping; Panel B).

**Table 4:** Association between available labor and female labor share, by production stage.

	Weeding				Field activities (all)			
	Share of total days: Household females		Share of total days: Hired females		Share of total days: Household females		Share of total days: Hired females	
	(1)	(2)	(3)	(4)	(5)	(6)	(7)	(8)
At least one migrant	0.008		−0.005		0.002		−0.003	
	(0.010)		(0.011)		(0.007)		(0.007)	
1 + male migrants		0.028**		−0.012		0.017**		−0.010
		(0.010)		(0.012)		(0.007)		(0.008)
1 + female migrants		−0.010		0.003		−0.008		0.004
		(0.012)		(0.009)		(0.008)		(0.005)
Difficulty finding labor	−0.004	−0.004	0.012	0.011	0.003	0.003	0.003	0.003
	(0.004)	(0.004)	(0.009)	(0.009)	(0.003)	(0.003)	(0.004)	(0.004)
Mean outcome (2017)	0.03	0.03	0.06	0.06	0.02	0.02	0.03	0.03
Mean outcome (2018)	0.05	0.05	0.03	0.03	0.03	0.03	0.02	0.02
p-value: Male = Female		0.013		0.124		0.019		0.052
Observations	1410	1410	1410	1410	1410	1410	1410	1410

	Stripping				Post field activities (all)			
	Share of total days: Household females		Share of total days: Hired females		Share of total days: Household females		Share of total days: Hired females	
	(9)	(10)	(11)	(12)	(13)	(14)	(15)	(16)

At least one migrant	0.010		−0.026		0.026**		−0.031**	
	(0.016)		(0.024)		(0.012)		(0.013)	
1 + male migrants		0.020		−0.032		0.024		−0.033*
		(0.022)		(0.032)		(0.015)		(0.017)
1 + female migrants		−0.007		−0.003		0.018		−0.017
		(0.024)		(0.028)		(0.018)		(0.017)
Difficulty finding labor	−0.006	−0.006	−0.025	−0.026	0.011	0.012	−0.007	−0.007
	(0.010)	(0.010)	(0.016)	(0.016)	(0.007)	(0.007)	(0.012)	(0.012)
Mean outcome (2017)	0.14	0.14	0.27	0.27	0.19	0.19	0.16	0.16
Mean outcome (2018)	0.19	0.19	0.15	0.15	0.23	0.23	0.07	0.07
p-value: Male = Female		0.457		0.555		0.805		0.534
Observations	1410	1410	1410	1410	1410	1410	1410	1410

Notes: Dependent variable is share of total labor days by worker type, for the specified activity or group of activities. Ordinary least squares regression using pooled sample with household-level fixed-effects. Controls are included for the area cultivated with jute by the household, and an indicator variable that takes the value 1 if an observation is of an ‘additional’ sample household in 2017, and zero otherwise. Standard errors are clustered by village. *,**, *** refer to significance at the 10%, 5% and 1% level respectively.

For field activities, we do not observe a significant change in the female share of jute labor when a household member moves out, regardless of whether we focus on household or hired labor. Thus, changes in labor allocations for field activities do not account for the finding that households with migrant members replace female hired labor by female household labor. However, coefficient estimates from columns (2) and (6) show this finding is because households with female migrants do not appear to change their labor allocations for field activities; male household member migration is associated with the use of more female household labor, consistent with the estimates in Table 3. For field activities, the share of female household labor is higher in households with male migrants than households with female migrants (*p* = 0.019). Thus, in households from which male members leave (who previously worked in the fields), female members may take over some of the field work, but in households from which female members leave, we do not see a similar response. Thus, the gender of the migrant appears to matter for field activities, where female labor is used relatively little. Households do not face a reduction in available household labor supply for field activities when female household members move out.

For post-field activities, in which women are traditionally more engaged, changes in the gender composition of labor are not significantly different for households with male versus female members: we consistently fail to reject the null hypothesis that the coefficients on having at least one male migrant or at least one female migrant are equal (*p* = 0.805 and *p* = 0.534 in Columns (14) and (16), respectively). For post-harvest activities, female outmigration could be associated with production more noticeably than for field activities, as these tasks are often done by women. This combination of findings suggests that although male and female household labor for homestead activities can be treated as substitutes, and although households could in principle turn to hired labor for these activities, female household members contribute disproportionally more time when the household’s labor supply has reduced, independent of the migrant’s gender.

Turning to the relative returns to working in jute production for men versus women, Table 5 considers associations between wages and migration. As migration reduces the supply of labor in a household, or as a household is experiencing difficulties finding labor, one might expect both male and female wages to increase. If female wages were to increase more than male wages, outmigration could help improve women’s benefits from participating in the jute value chain by reducing prevailing gender wage gaps. However, we do not find a statistically significant difference in hourly wages paid to either male laborers in Columns (1) and (2), or female laborers in Columns (3) and (4). As a result, we find no evidence of outmigration or perceived labor scarcity being associated with changes to the gender gap in casual labor wages.

**Table 5:** Association between available labor, expenditure and wages for hired labor.

Hourly wage (USD), by gender						
	Male hired laborers		Female hired laborers		Wage gap (Male - Female)	
	(1)	(2)	(3)	(4)	(5)	(6)
At least one migrant	−0.016		−0.002		−0.024	
	(0.030)		(0.034)		(0.071)	
1 + male migrants		−0.005		−0.015		0.058
		(0.033)		(0.038)		(0.060)
1 + female migrants		−0.014		0.049		−0.089
		(0.038)		(0.048)		(0.130)
Difficulty finding labor	−0.034	−0.034	−0.022	−0.020	0.034	0.033
	(0.038)	(0.038)	(0.024)	(0.024)	(0.045)	(0.044)
Mean outcome (2017)	0.61	0.61	0.37	0.37	0.25	0.25
Mean outcome (2018)	0.85	0.85	0.48	0.48	0.40	0.40
p-value: Male = Female		0.856		0.328		0.285
Observations	1387	1387	905	905	887	887

Notes: Ordinary least squares regression using pooled sample with household-level fixed-effects. Controls are included for the area cultivated with jute by the household, and an indicator variable that takes the value 1 if an observation is of an ‘additional’ sample household in 2017, and zero otherwise. Standard errors are clustered by village. *,**, *** refer to significance at the 10%, 5% and 1% level respectively. Expenditure outcomes are reported for all households (and take the value zero if no hired labor). Wage variables are conditional on reporting at least one laborer, the difference in wages is restricted to households reporting hiring at least one male and one female laborer.

Finally, we explore whether changes in labor availability are associated with changes in empowerment outcomes, using our additional sample, for whom we collected pro-WEAI data in both 2017 and 2018. Table 6 presents estimates of the model in Eqs. (3), (4), using both an aggregate indicator for migration (odd-numbered specifications) and an indicator disaggregated by migrant gender (even-numbered specifications).

**Table 6:** Association between female empowerment outcomes and migration, additional sample.

	Male empowered		Male score		Female empowered		Female score		Intra-HH inequality	
	(1)	(2)	(3)	(4)	(5)	(6)	(7)	(8)	(9)	(10)
At least one migrant	0.010		0.010		0.094*		0.035**		0.016	
	(0.060)		(0.013)		(0.049)		(0.015)		(0.021)	
At least one male migrant		0.027		0.012		−0.083		−0.030		−0.044
		(0.089)		(0.015)		(0.052)		(0.020)		(0.027)
At least one female migrant		−0.055		−0.003		0.199***		0.056**		0.046
		(0.070)		(0.018)		(0.074)		(0.023)		(0.028)
Change in difficulty finding labor (2018–2017)	0.027	0.028	0.008	0.008	0.022	0.022	0.013	0.013	0.018	0.018
	(0.037)	(0.037)	(0.010)	(0.010)	(0.032)	(0.031)	(0.012)	(0.011)	(0.011)	(0.011)
Outcome variable (2017)	0.130**	0.128**	0.117**	0.118**	0.128**	0.135**	0.300***	0.299***	0.142***	0.141***
	(0.050)	(0.051)	(0.045)	(0.045)	(0.055)	(0.055)	(0.048)	(0.048)	(0.043)	(0.043)
Observations	457	457	457	457	477	477	477	477	451	451

Notes: Ordinary least squares regression using empowerment sample reports for 2018. Standard errors are clustered by village. *,**, *** refer to significance at the 10%, 5% and 1% level respectively.

For male respondents, we find no significant association between changes in empowerment scores and migration by either male or female household members. In contrast, for female respondents, we observe a statistically significant and positive association between empowerment and when a household member moves out. Controlling for their empowerment status in 2017, female respondents in households with migrants are 9.4 percentage points more likely to be empowered in 2018 than respondents in households without migration; and these women’s empowerment scores (i.e. the proportion of sub-domains in which they are considered adequately empowered) increase by 3.5 percentage points. Changes are largest in households with female members moving out: when another woman leaves, female respondents are 20 percentage points more likely to be empowered relative to their peers. This change is not related to a respondent change; when we restrict our sample to households without change in respondent from 2017 to 2018, we find similar results (Appendix Table 2).

**Fig. 4:**
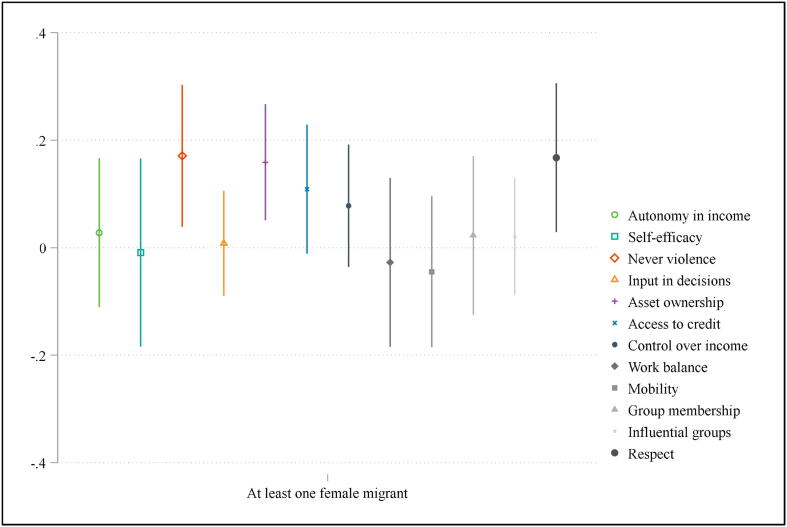
Regression estimates of disaggregated pro-WEAI outcomes on female migrant status.

In Fig. 4, we analyze which sub-domains are driving this result, using the same specification with gender-disaggregated migrant data, i.e. Eq. (4), but using as our outcome variables the indicator for being adequately empowered within each sub-domain of the pro-WEAI. Whereas women face an increased workload when other female household members move out, we find statistically significant improvements in empowerment across three sub-domains: attitudes to intimate-partner violence, asset ownership and respect among household members for households reporting one or more female members migrating.^15^ One potential hypothesis is that having multiple adult women within a household may lead to more competition over resources or relative standing, which migration alleviates. This interpretation is consistent with Raghunathan, Cunningham, Quisumbing, and Doss (2019), who find the empowerment of different women in the household relative to one another is an important determinant of household outcomes. These findings underscore that analyses on the relationship between intra-household dynamics and empowerment outcomes should move beyond interactions between husband and wife and take into consideration broader household structure.

## 5 Conclusion

In this paper, we describe changes in the gendered allocation of labor among smallholder jute-producing households in a context of widespread rural out-migration in southern Bangladesh. Based on collective household models, we posited four possible ways in which households may respond to changes in labor supply: family members taking on more agricultural tasks; hiring labor (though an imperfect substitute for household labor, and at increased wages as labor becomes scarce); substituting capital for labor, at least for tasks for which machine rental is possible; and reducing the area or intensity of cultivation. We add nuance to these predictions in a context where gender norms are closely linked to the division of tasks between male and female workers, and the compensation male and female workers each receive for completing those tasks.

Controlling for area cultivated, households predominantly appear to use female family labor instead of hired labor in reaction to changes in labor supply, but our results suggest that male and female labor are substitutes only to a very limited extent. Both out-migration of household members and scarcity of agricultural labor are associated with an increase in the share of labor for jute performed by female household members. However, the increased work by female household members is concentrated in post-harvest processing activities that take place in the compound, rather than throughout the production process in the field. Prevailing gender norms appear too important to overcome barriers for women to take on men’s tasks in the field, regardless of whether labor shortages are associated with out-migration of male family members or with general shortages of hired labor. Further, when female household members move out, households do not offset the reduced labor supply by hiring additional female workers, suggesting that especially female hired labor is an imperfect substitute for family labor.

We also do not find strong evidence to suggest that out-migration is welfare-enhancing for women who remain in agricultural households. Female household members are not necessarily rewarded for their added workload: women’s empowerment increases only when another female household member moves out, not when a male household member migrates. Moreover, migration of household members is not associated with an improvement in outcomes for female wage laborers, as we observe a small reduction in the use of female hired labor, and a persistent gap in wages paid to male versus female workers. Whilst one might consider outmigration of working-age adults and scarcity of rural wage labor as an opportunity to empower women, such gains may not be realized in the absence of deliberate interventions and gender-responsive programming to address prevailing norms and empowerment gaps.

Another policy implication relates to agricultural technology adoption, as capital can act as a substitute for labor in agricultural production. Introducing labor-saving technologies can influence welfare outcomes differently for men versus women (e.g. Afridi, Bishnu, & Mahajan, 2020), including in the context that we studied, where we would anticipate that dynamics around the introduction of labor saving technologies would depend on whether they target activities typically undertaken by men, women, or both genders. Had we not collected data disaggregated by both gender and task, we would have been unable to observe to what extent male and female labor act as substitutes for one another. These findings underscore the value of collecting gender-and task-disaggregated data on labor in the agricultural production process, and we believe that it is worth extending such detailed data collection to other similar contexts in which men and women collaborate in agricultural production, to learn more about how gendered divisions of different tasks within crop production can be influenced by external factors such as increasing migrant opportunity, and how this, in turn, could influence the relative benefits of labor-saving technologies for men versus women in producer households and agricultural labor markets.

Finally, the paper leverages the gender- and task-disaggregated labor modules by linking these findings to panel data that allow documenting changes in women’s (and men’s) empowerment in agriculture. Our analysis does find that household female respondents report greater intrinsic agency following out-migration of female household members. We do not find this result when male household members move out, even though male and female outmigration are associated with a similar increase in the relative contribution of female family labor to production. This demonstrates the potential importance of intra-household dynamics for empowerment across the entire household– including between women– not just between spouses. An important policy implication arising from this result is that gender-responsive programming should look beyond couples in order to address empowerment gaps within a family.

In conclusion, while gender roles in agriculture may evolve with increased migration, our findings indicate that this process does not happen rapidly. In traditional societies like the southern delta region of Bangladesh, gender segregation of tasks continues to be the norm, and gaps in labor use, wages, and empowerment persist, despite the labor shortages that households face as their members, and laborers from their communities, seek their fortune by migrating to urban settings. Better understanding the nuances of these labor dynamics and identifying how they relate to gender gaps in empowerment, through analyzing gender- and task-disaggregated data, is key for inclusive agricultural development.

## CRediT authorship contribution statement

**Alan de Brauw:** Conceptualization, Supervision, Formal analysis, Writing - original draft, Writing - review & editing, Project administration, Funding acquisition, Investigation. **Berber Kramer:** Conceptualization, Supervision, Formal analysis, Writing - original draft, Writing - review & editing, Project administration, Funding acquisition, Investigation. **Mike Murphy:** Conceptualization, Supervision, Formal analysis, Writing - original draft, Writing - review & editing, Project administration, Investigation, Data curation, Validation.

## Declaration of Competing Interest

The authors declare that they have no known competing financial interests or personal relationships that could have appeared to influence the work reported in this paper.

## Figures and Tables

**Table A1:** Summary statistics for primary analysis variables.

	2017 Sample					2018 Sample				
	Mean	SD	Min	Max	N	Mean	SD	Min	Max	N
***Migration & labor scarcity***										
At least one migrant	0.10	0.30	0.00	1.00	1410	0.20	0.40	0.00	1.00	1410
At least one male migrant	0.07	0.25	0.00	1.00	1410	0.12	0.32	0.00	1.00	1410
At least one female migrant	0.06	0.23	0.00	1.00	1410	0.11	0.32	0.00	1.00	1410
Difficulty finding labor	0.45	0.50	0.00	1.00	1410	0.38	0.48	0.00	1.00	1410

***Labor usage***										
Used male household labor	0.98	0.14	0.00	1.00	1410	0.99	0.11	0.00	1.00	1410
Used female household labor	0.86	0.34	0.00	1.00	1410	0.95	0.22	0.00	1.00	1410
Used male hired labor	0.96	0.21	0.00	1.00	1410	0.95	0.21	0.00	1.00	1410
Used female hired labor	0.53	0.50	0.00	1.00	1410	0.45	0.50	0.00	1.00	1410
Share of labor days: Female (Household)	0.11	0.10	0.00	0.82	1409	0.12	0.10	0.00	0.69	1409
Share of labor days: Female (Hired)	0.10	0.13	0.00	0.66	1409	0.04	0.08	0.00	0.52	1409
Share of labor days: Female	0.21	0.16	0.00	0.85	1409	0.17	0.12	0.00	0.74	1409

***Wages***										
Hourly wage: Male	0.59	0.14	0.28	2.13	1348	0.75	0.27	0.01	5.00	1343
Hourly wage: Female	0.37	0.15	0.08	1.88	737	0.48	0.21	0.08	2.66	623
Hourly wage difference (Male - Female)	0.24	0.19	−1.15	1.71	726	0.28	0.30	−1.97	3.38	611

***Empowerment outcomes***										
Respondent empowered: Male	0.36	0.48	0.00	1.00	240	0.44	0.50	0.00	1.00	460
Empowerment score: Male	0.66	0.14	0.33	1.00	240	0.69	0.12	0.33	1.00	460
Respondent empowered: Female	0.17	0.37	0.00	1.00	240	0.21	0.41	0.00	1.00	480
Empowerment score: Female	0.53	0.19	0.00	1.00	240	0.57	0.16	0.08	1.00	480
Intra-household inequality	−0.13	0.21	−0.75	0.50	237	−0.12	0.19	−0.75	0.33	457

**Table A2:** Association between female empowerment outcomes and migration, additional sample.

	Male empowered		Male score		Female empowered		Female score		Female - male score	
	(1)	(2)	(3)	(4)	(5)	(6)	(7)	(8)	(9)	(10)
At least one migrant	0.015		0.010		0.106**		0.036**		0.020	
	(0.062)		(0.013)		(0.052)		(0.016)		(0.022)	
At least one male migrant		0.032		0.013		−0.075		−0.027		−0.048*
		(0.088)		(0.015)		(0.056)		(0.021)		(0.028)
At least one female migrant		−0.059		−0.006		0.211***		0.055**		0.056*
		(0.072)		(0.019)		(0.075)		(0.022)		(0.029)
Difficulty finding labor (2018–2017)	0.030	0.032	0.007	0.008	0.030	0.027	0.013	0.013	0.017	0.016
	(0.037)	(0.037)	(0.010)	(0.010)	(0.033)	(0.032)	(0.012)	(0.012)	(0.011)	(0.011)
Outcome variable (2017)	0.126**	0.125**	0.119**	0.120**	0.126**	0.134**	0.303***	0.303***	0.146***	0.145***
	(0.052)	(0.053)	(0.046)	(0.046)	(0.055)	(0.054)	(0.047)	(0.047)	(0.045)	(0.044)
Observations	444	444	444	444	459	459	459	459	439	439

Notes: Ordinary least squares regression using empowerment sample reports for 2018. Standard errors are clustered by village. *,**, *** refer to significance at the 10%, 5% and 1% level respectively.

**Table A3:** Association between available labor and female labor share.

	Share of total days by household females	Share of total days by hired females	Share of total days by females
	(1)	(2)	(3)
Alternative migrant indicator	0.022**	−0.027***	−0.005
	(0.008)	(0.010)	(0.012)
Difficulty finding labor	0.010**	0.008	0.018**
	(0.004)	(0.007)	(0.007)
Mean outcome (2017)	0.11	0.10	0.21
Mean outcome (2018)	0.12	0.04	0.17
Observations	1410	1410	1410

Notes: Ordinary least squares regression using pooled sample with household-level fixed-effects.

Standard errors are clustered by village. *,**, *** refer to significance at the 10%, 5% and 1% level respectively.

**Table A4:** Association between available labor and female labor share, by production stage.

	Weeding		Field activities (all)		Stripping		Post field activities (all)	
	Total share days HH female	Total share days HH female	Total share days HH female	Total share days HH female	Total share days HH female	Total share days HH female	Total share days HH female	Total share days HH female
	(1)	(2)	(3)	(4)	(5)	(6)	(7)	(8)
Alternative migrant indicator	0.002	−0.011	0.003	−0.007	0.035**	−0.045*	0.037***	−0.045***
	(0.013)	(0.010)	(0.008)	(0.006)	(0.016)	(0.024)	(0.011)	(0.016)
Difficulty finding labor	−0.005	0.013	0.003	0.004	−0.011	−0.010	0.007	0.002
	(0.004)	(0.009)	(0.003)	(0.004)	(0.010)	(0.019)	(0.007)	(0.013)
Mean outcome (2017)	0.03	0.06	0.02	0.03	0.14	0.27	0.19	0.16
Mean outcome (2018)	0.05	0.03	0.03	0.02	0.19	0.15	0.23	0.07
Observations	1410	1410	1410	1410	1410	1410	1410	1410

Notes: Ordinary least squares regression using pooled sample with household-level fixed-effects. Standard errors are clustered by village. *,**, *** refer to significance at the 10%, 5% and 1% level respectively.

**Table A5:** Association between available labor, expenditure and wages for hired labor\

	Hourly wage: male laborers (USD)	Hourly wage: female laborers (USD)	Male wage - female wage (USD)
	(1)	(2)	(3)
Alternative migrant indicator	0.024	0.006	0.010
	(0.022)	(0.027)	(0.052)
Difficulty finding labor	−0.050	−0.018	0.040
	(0.042)	(0.024)	(0.046)
Mean outcome (2017)	0.61	0.37	0.25
Mean outcome (2018)	0.85	0.48	0.40
Observations	1387	905	887

Notes: Ordinary least squares regression using pooled sample with household-level fixed-effects.

Standard errors are clustered by village. *,**, *** refer to significance at the 10%, 5% and 1% level respectively.

**Table A6:** Association between female empowerment outcomes and migration, additional sample.

	Male empowered	Male score	Female empowered	Female score	Female - male score
	(1)	(2)	(3)	(4)	(5)
At least one migrant	−0.010	0.009	0.072	0.032**	0.014
	(0.063)	(0.013)	(0.049)	(0.016)	(0.022)
Difficulty finding labor (2018–2017)	0.028	0.008	0.021	0.012	0.018
	(0.037)	(0.010)	(0.032)	(0.012)	(0.011)
Outcome variable (2017)	0.131**	0.117**	0.129**	0.302***	0.143***
	(0.050)	(0.045)	(0.055)	(0.048)	(0.043)
Observations	457	457	477	477	451

Notes: Ordinary least squares regression using empowerment sample reports for 2018. Standard errors are clustered by village. *,**, *** refer to significance at the 10%, 5% and 1% level respectively.

**Table A7:** Association between available labor, expenditure and wages for hired labor.

	Hourly wage: male laborers (USD)	Hourly wage: male laborers (USD)	Hourly wage: female laborers (USD)	Hourly wage: female laborers (USD)	Male wage - female wage (USD)	Male wage - female wage (USD)
	(1)	(2)	(3)	(4)	(5)	(6)
At least one migrant	0.066		0.030		0.036	
	(0.054)		(0.030)		(0.067)	
At least one male migrant		0.122**		0.017		0.105*
		(0.052)		(0.035)		(0.061)
At least one female migrant		0.015		0.065		−0.050
		(0.118)		(0.051)		(0.133)
Difficulty finding labor	0.020	0.020	−0.017	−0.016	0.037	0.037
	(0.045)	(0.044)	(0.025)	(0.024)	(0.046)	(0.045)
Mean outcome (2017)	0.61	0.61	0.37	0.37	0.25	0.25
Mean outcome (2018)	0.85	0.85	0.48	0.48	0.40	0.40
p-value: Male = Female		0.397		0.491		0.303
Observations	887	887	887	887	887	887

Notes: Ordinary least squares regression using pooled sample with household-level fixed-effects. Standard errors are clustered by village. *,**, *** refer to significance at the 10%, 5% and 1% level respectively.

## Appendix Tables

Alternative specification (Table 5) restricted to households with the same respondents across rounds

Aggregated migrant specifications with alternative migrant report

Wages regression restricted to households reporting both male and female labor

## Footnotes

1 This individual was identified based on the household report at the time of the first interview. If this individual left the household or was otherwise unable to continue as the respondent in subsequent interviews, another household member acted as respondent. In practice, this was rare, with 97% of households using the same primary respondent for the 2017 and 2018 interviews.

2 The former is more prevalent in our context. The latter– known as ‘white jute’– is generally regarded as producing finer quality fibers.

3 See Ali (2012) for a comprehensive treatment of the historical and political role of jute in Bangladesh.

4 Since 2017, exports have been subject to an anti-dumping duty in India, which is a major market for Bangladeshi jute. We cannot rule out that this policy change may have induced additional migration in our sample, as individuals may have been less likely to migrate under more favorable macroeconomic conditions.

5 For the 2017 survey the median area was 84 decimals (0.34 ha).

6 Slightly more restrictive criteria were used for newly sampled households: they were required to have at least ten years of experience farming jute, and to have a minimum landholding of 66 decimals (0.27 ha).

7 Summary statistics for the analysis variables described in this section are provided in Appendix Table 1.

8 Note that for the additional sample we do not have this information for the 2017 round, since they did not complete a 2016 interview. Therefore, we assign a value of zero to them for the 2017 round. We include an alternative definition based on a retrospective report for which we have data for this sample as a robustness check, and find similar results (Appendix Table 3, Table 4, Table 5, Table 6).

9 Female migrants predominantly report migrating permanently (approximately 80% of female migrants) whereas male migrants are more likely to report migrating for short-term employment reasons.

10 To prevent reporting errors from influencing results, we winsorize all continuous labor measures at the 1st/99th percentile.

11 These sub-domains are: autonomy in income; self-efficacy; attitudes about intimate partner violence; respect among household members (intrinsic agency); input in productive decisions; ownership of land and other assets; access to and decisions on financial services; control over use of income; work balance; mobility (instrumental agency); group membership, and membership in influential groups (collective agency).

12 The definition of adequacy within each sub-domain is described in Malapit et al. (2019).

13 Since we have two periods, by the Frisch-Waugh theorem the estimator is equivalent to first differencing.

14 For annualized outcomes, *Scarcity* takes the value 1 if difficulty is reported for at least one activity within the survey period.

15 Measurement properties of these sub-indices are discussed in Yount et al. (2019).
